# Post‐hoc analysis investigating the safety and efficacy of brexpiprazole in Japanese patients with schizophrenia who were switched from other antipsychotics in a long‐term study (Secondary Publication)

**DOI:** 10.1002/npr2.12107

**Published:** 2020-04-15

**Authors:** Jun Ishigooka, Tomohiro Usami, Shuichi Iwashita, Yoshitsugu Kojima, Satoshi Matsuo

**Affiliations:** ^1^ Tokyo Women’s Medical University Tokyo Japan; ^2^ Medical Affairs Otsuka Pharmaceutical Co., Ltd. Tokyo Japan; ^3^ Headquarters of Clinical Development Otsuka Pharmaceutical Co., Ltd. Osaka Japan; ^4^ Medical Affairs Otsuka Pharmaceutical Co., Ltd. Osaka Japan

**Keywords:** antipsychotics, brexpiprazole, dopamine partial agonist, schizophrenia, switching

## Abstract

A post hoc analysis was performed using data obtained over eight weeks from 200 Japanese patients with schizophrenia who were switched to brexpiprazole monotherapy in a long‐term treatment study. The 8‐week period comprised of a 4‐week switching phase and a 4‐week post‐switch phase. For the antipsychotic switching schedule, brexpiprazole was first administered at 1 mg/day and increased to 2 mg/day by the end of week 4. Concurrently, the previous antipsychotic(s) was/were tapered gradually from the start of week 3 and discontinued by the end of week 4. Brexpiprazole could then be increased up to 4 mg/day according to the CGI‐I criteria. At week 8, 1.8%, 23.2%, 25.0%, and 50% of patients were administered daily brexpiprazole doses of 1, 2, 3, and 4 mg, respectively. The discontinuation rate at week 8 was 17.0%. The major reasons for discontinuation were consent withdrawal (9.5%), occurrence of adverse events (5.5%), and physician's decision (2.0%). Commonly reported adverse events were nasopharyngitis (13.5%), schizophrenia (9.0%), insomnia (6.5%), headache (5.5%), and akathisia (5.5%). The discontinuation rate was 4.9% for patients who were switched from aripiprazole as the primary antipsychotic and 25.4% for those who were switched from other antipsychotics. Owing to the serious adverse events that led to treatment discontinuation, careful switching to brexpiprazole is necessary in patients who previously used olanzapine as their primary antipsychotic.

## Abstract 2

A post hoc analysis was performed using data obtained over eight weeks from 200 Japanese patients with schizophrenia who were switched to brexpiprazole monotherapy in a long‐term treatment study. The 8‐week period comprised of a 4‐week switching phase and a 4‐week post‐switch phase. The discontinuation rate was 4.9% for patients who were switched from aripiprazole as the primary antipsychotic and 25.4% for those who were switched from other antipsychotics.

## Graphical abstract

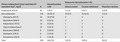

## 1 INTRODUCTION

Brexpiprazole is a novel antipsychotic that was approved in Japan for the treatment of schizophrenia in January 2018. Pharmacologically, it is a partial agonist with high affinity for the dopamine D_2_ receptor and serotonin 5HT_1A_ receptor, and an antagonist with high affinity for the 5HT_2A_ receptor. Unlike for aripiprazole, which belongs to the same drug class as brexpiprazole, a lower intrinsic potency on the dopamine D_2_ receptor and a higher affinity for the serotonin receptors were found for brexpiprazole.^1^ These pharmacological properties are expected to yield a favorable antipsychotic effect and reduce the risk of adverse events that are commonly observed with typical or atypical antipsychotics (eg, extrapyramidal symptoms, akathisia, insomnia, and sedation).^2^

In the treatment of psychiatric disorders such as schizophrenia, antipsychotic switches are routinely used to obtain better therapeutic responses with fewer side effects, or to reduce drug‐related secondary negative symptoms or cognitive impairment by simplifying treatment regimens (reduced number of drugs and reduced doses) for which is used to reduce/manage the side effects.^3^ However, antipsychotic switches may be associated with the withdrawal phenomena due to drug discontinuation or dose reduction of the previous antipsychotic(s), adverse events, insufficient effects, or deteriorated psychiatric symptoms attributable to replacement of new antipsychotics. Accordingly, antipsychotic switches should be performed with great caution.^3^

Although there are recommended guidelines for switching for some antipsychotics, more experience is required in clinical practice to reach a consensus that is sufficient to establish guidelines. Particularly for antipsychotics that are new in the market, careful evaluations of the data retrieved in clinical practice are important.^4^, ^5^, ^6^ Here, we performed an exploratory post hoc analysis with Japanese patients who were switched from previous antipsychotics to the novel antipsychotic brexpiprazole in a long‐term treatment study to assess the outcomes when a patient's previous antipsychotic was switched to brexpiprazole.

## 2 METHODS

### 2.1 Clinical study

The target population of the study was de novo patients who were eligible for a switch to brexpiprazole monotherapy and patients who had completed a placebo‐controlled, double‐blind, short‐term treatment study with brexpiprazole. The long‐term treatment study was conducted in compliance with the International Conference on Harmonisation Good Clinical Practice guidelines and Japanese Ministerial Ordinance on Good Clinical Practice. Each study site initiated patient enrollment after their institutional review boards (IRBs) reviewed and approved the study. All patients provided written informed consent before participation in the study. Written consent for minors was obtained from their legal representatives (parental authorities, spouses, guardians, or any person equivalent to them), but a personal signed informed consent was also provided by these patients. The results of the long‐term treatment study have been published by Ishigooka et al.^7^

### 2.2 Study population and assessment period

The post hoc analysis was on de novo patients who were switched to brexpiprazole monotherapy in this long‐term treatment study.^7^ Since this post hoc analysis aimed to assess the influence of switches to brexpiprazole monotherapy, patients who did not use antipsychotics 30 days before informed consent retrieval and those who were withdrawn from the study due to deviations from the protocol were excluded. The assessment period was 8 weeks, which was composed of a 4‐week protocol‐defined switching phase and a 4‐week post‐switch phase, during which the influence of the switch to brexpiprazole monotherapy was observed.

### 2.3 Antipsychotic switching procedure and the doses of brexpiprazole

**FIGURE 1:**
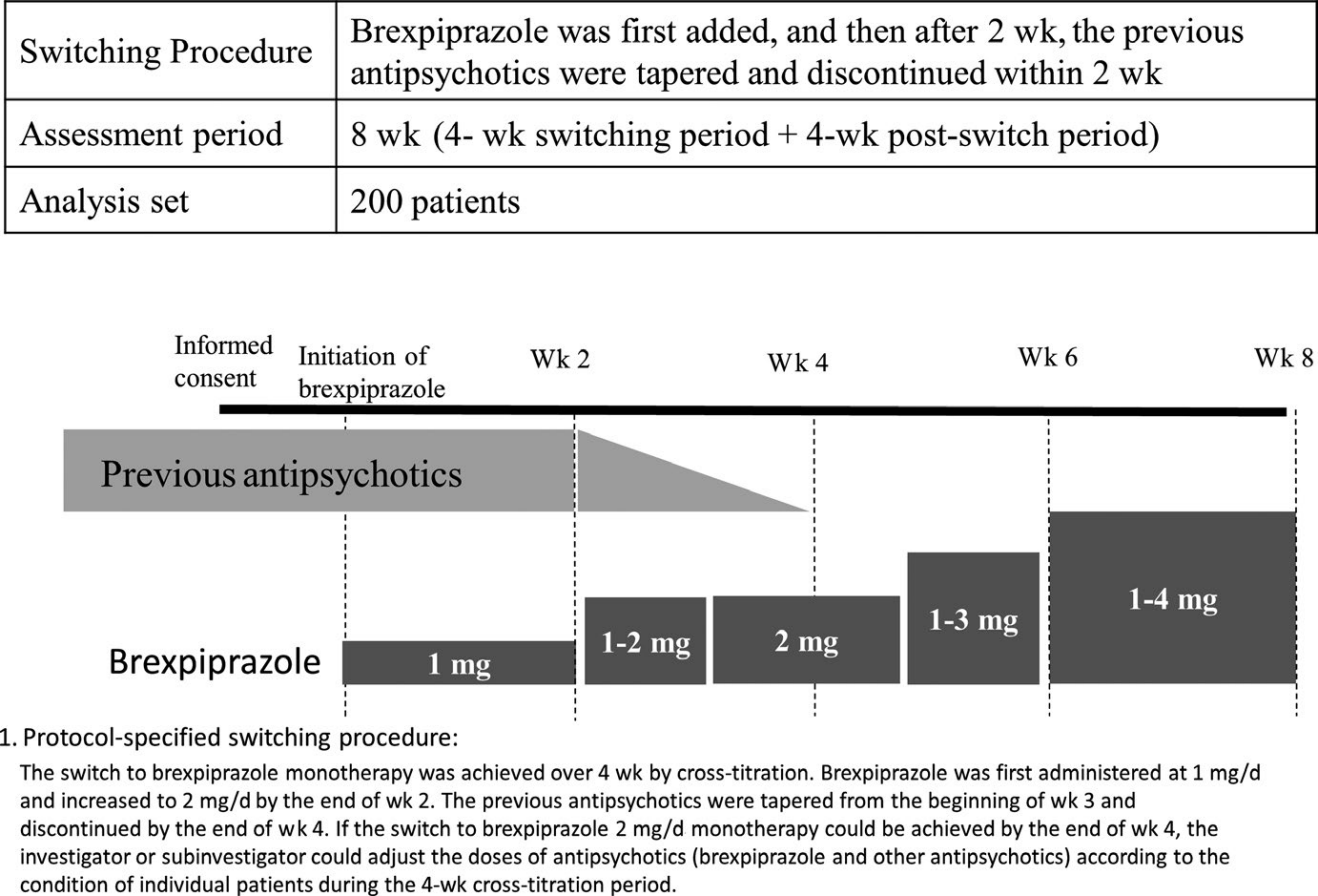
Switching procedure and analysis set

The switch to brexpiprazole monotherapy was achieved by cross‐titration over 4 weeks, that is, brexpiprazole was administered alongside the previous antipsychotic(s) for the first 2 weeks, followed by tapering of the previous antipsychotic(s) and, ultimately, discontinuation of the previous drug(s) in the latter 2 weeks. The initial dose of brexpiprazole was 1 mg/day, and if tolerable, it was increased to 2 mg/day by the end of week 4. After week 5 (1 week after switching was successfully achieved), brexpiprazole was increased up to 4 mg/day according to the Clinical Global Impression‐Improvement (CGI‐I) criteria, if no issues with tolerability occurred. The previous antipsychotic(s) were gradually tapered over 2 weeks from the beginning of week 3 and discontinued by the end of week 4 (Figure 1).

### 2.4 Prior antipsychotic and antiparkinsonian treatments

In this post hoc analysis, antipsychotics that were used 30 days before informed consent retrieval were defined as previous antipsychotics to reflect the treatments provided to each patient before enrollment in this study. The doses of the previous antipsychotics were converted to chlorpromazine (CP)‐equivalent doses.^8^, ^9^ Parenterals, as‐needed drugs, and vegetamin (a combination product, domestic medicine in Japan) were excluded from CP‐equivalent doses. The primary antipsychotic for each patient was defined as an antipsychotic that was administered at the highest CP‐equivalent dose 30 days before informed consent retrieval. Similarly, the doses of antiparkinsonian drugs administered 30 days before informed consent retrieval were converted to biperiden‐equivalent doses.^8^

### 2.5 Analysis methods

Patient characteristics (age, age at onset, duration of disease, baseline body weight, baseline body mass index [BMI], baseline Positive and Negative Syndrome Scale [PANSS] total score, and baseline CGI‐S score) were summarized using descriptive statistics. The use of antipsychotic(s) 30 days before informed consent retrieval (primary antipsychotic(s), number of antipsychotics, total daily CP‐equivalent dose of antipsychotics, and total daily CP‐equivalent dose of antipsychotics by primary antipsychotic) was summarized using frequencies (number and percentage of patients) and descriptive statistics. The use of antiparkinsonian drugs administered 30 days before informed consent retrieval (number of antiparkinsonian drugs and the daily total biperiden‐equivalent dose of the drugs) was summarized using frequencies (number and percentage of patients) and/or descriptive statistics. Baseline data were values observed before the antipsychotic switch (ie, before starting brexpiprazole therapy).

The numbers and percentages of patients exposed to brexpiprazole were summarized by daily dose at the start of brexpiprazole and every 2 weeks thereafter.

The numbers and percentages of patients who discontinued or completed treatment during the 4‐week switching phase, 4‐week post‐switch phase, and 8‐week assessment period (4‐week switching phase + 4‐week post‐switch phase) were calculated. The number and percentage of patients who discontinued treatment were summarized by reason for discontinuation.

The numbers and percentages of patients who discontinued or completed treatment over the 8‐week assessment period (4‐week switching phase + 4‐week post‐switch phase) were categorized based on patient characteristics and primary antipsychotic(s). The number and percentage of patients who discontinued treatment were summarized by primary antipsychotic(s) and reason for discontinuation. For patients who completed or discontinued treatment, patient characteristics and total daily CP‐equivalent dose of the antipsychotic(s) administered 30 days before informed consent retrieval were summarized separately using descriptive statistics.

We performed univariate logistic regression with treatment discontinuation or completion over the 8‐week assessment period (4‐week switching phase + 4‐week post‐switch phase) using continuous variables of age, baseline PANSS total score, number of previous antipsychotics, and the total daily CP‐equivalent dose of prior antipsychotics, as well as the type of primary antipsychotic(s) (aripiprazole or non‐aripiprazole, olanzapine or non‐olanzapine, risperidone or non‐risperidone, and paliperidone or non‐paliperidone) as explanatory variables. For each parameter, an estimate, standard error, Wald test *P* value, and odds ratio with its 95% confidence interval (two‐sided) were determined. No significance test was performed, and *P* values were presented for reference.

Adverse events with an incidence of ≥ 5%, serious adverse events, and adverse events leading to treatment discontinuation during the 4‐week switching phase, the 4‐week post‐switch phase, and the 8‐week assessment period (4‐week switching phase + 4‐week post‐switch phase) were summarized using frequencies (number and percentage of patients). Adverse events that led to the discontinuation of brexpiprazole therapy are listed by the day of onset, causal relationship to the trial drug, severity, and seriousness.

For PANSS total score, changes from baseline over the 8‐week assessment period (4‐week switching phase + 4‐week post‐switch phase) were summarized using descriptive statistics. Similar statistical analysis was performed for patients who used aripiprazole or non‐aripiprazole antipsychotics as primary antipsychotic. Imputations were not conducted for missing data.

## 3 RESULTS

### 3.1 Patient disposition and characteristics

Among 208 Japanese patients who were switched to brexpiprazole monotherapy in a long‐term treatment study, two patients did not use antipsychotics 30 days before providing informed consent and six were withdrawn from the analysis due to protocol deviations. The post hoc analysis was therefore performed with the data retrieved from the remaining 200 patients.

Patient characteristics (mean [standard deviation, SD]) at the time of informed consent retrieval were as follows: age, 45.7 (15.1) years old; duration of disease, 17.1 (14.2) years; PANSS total score, 69.8 (22.1); and total daily CP‐equivalent dose of the antipsychotic administered 30 days before informed consent retrieval, 482.6 (359.8) mg/day (Table 1). Of the 200 patients, 156 (78.0%) used one antipsychotic, 34 (17.0%) used two antipsychotics, and 10 (5.0%) used ≥ 3 antipsychotics 30 days before providing informed consent. As their primary antipsychotic, 186 patients (93.0%) used atypical antipsychotics: 82 patients (41.0%), 36 patients (18.0%), and 33 patients (16.5%) used aripiprazole, olanzapine, and risperidone, respectively.

**TABLE 1:** Characteristics of patients who completed or discontinued treatment

	Completers n = 166	Discontinuations n = 34	Total n = 200
Age (y)	46.1 (14.9) [21‐79]	44.0 (15.7) [18‐76]	45.7 (15.1) [18‐79]
Age of onset (y)	29.0 (10.2) [13‐57]	26.7 (11.4) [14‐75]	28.6 (10.4) [13‐75]
Duration of disease (y)	17.0 (14.1) [0‐55]	17.3 (14.9) [1‐52]	17.1 (14.2) [0‐55]
Body weight (kg)	65.1 (14.2) [33.0‐110.6]	64.6 (15.3) [39.9‐96.2]	65.0 (14.4) [33.0‐110.6]
BMI (kg/m^2^)	24.4 (4.4) [14.5‐43.4]	24.9 (4.9) [17.6‐35.9]	24.5 (4.4) [14.5‐43.4]
PANSS total score	69.6 (21.6) [33.0‐129.0]	70.4 (24.8) [32.0‐127.0]	69.8 (22.1) [32.0‐129.0]
CGI‐S	3.4 (1.1)〔1‐6〕	3.6 (1.2)〔1‐6〕	3.4 (1.1)〔1‐6〕
Total daily CP‐equivalent dose of prior antipsychotics (mg/d)	472.8 (333.1) [25‐1703]	530.3 (472.5) [75‐2406]	482.6 (359.8) [25‐2406]

Note

Data are expressed as mean (SD) [minimum‐maximum].

Abbreviations: BMI, body mass index; CGI‐S, Clinical Global Impression‐Severity of illness; CP, chlorpromazine; PANSS, Positive and Negative Syndrome Scale; SD, standard deviation.

### 3.2 Exposure to brexpiprazole

Patients receiving the respective daily brexpiprazole doses of 1 mg and 2 mg were 74.7% and 25.3% at week 2, respectively; and 5.9% and 94.1% at week 4, respectively. At week 6, 0.6%, 32.0%, 61.7%, and 5.7% of patients received 1 mg, 2 mg, 3 mg, and 4 mg, respectively, while at week 8, 1.8%, 23.2%, 25.0%, and 50.0% of patients received 1 mg, 2 mg, 3 mg, and 4 mg, respectively.

### 3.3 Use of antiparkinsonian drugs

The proportion of patients receiving antiparkinsonian drugs 30 days before informed consent retrieval was 29.0% (58 of 200 patients), and this proportion decreased to 12.4% (21 of 170 patients) at week 8 (ie, 4 weeks after the switch). For the mean daily biperiden‐equivalent dose of the antiparkinsonian drugs, a similar value was found at 30 days before informed consent retrieval (2.7 mg/day) and week 8 (ie, 4 weeks after the switch; 2.8 mg/day).

### 3.4 “Discontinuations” and “completers”

The discontinuation rate over the eight weeks was 17.0%. The major causes of discontinuation were consent withdrawal (9.5%), adverse events (5.5%), and physician's discretion (2.0%). The discontinuation rate was 9.5% during the 4‐week switching phase (19 of 200 patients) and 8.3% during the 4‐week post‐switch phase (15 of 181 patients) (Table 2). Comparisons of patient characteristics between “discontinuations” and “completers” (Table 1) revealed that the total daily CP‐equivalent dose for the antipsychotics taken 30 days before informed consent retrieval was approximately 60 mg higher in “discontinuations” (530.3 mg) than in “completers” (472.8 mg/day). However, the total daily CP‐equivalent dose of previous antipsychotics exceeded 2400 mg in one patient who discontinued treatment.

**TABLE 2:** Patient disposition

	4‐wk switching period n (%)	4‐wk post‐switch period n (%)	8‐wk assessment period n (%)
Completers	181 (90.5)	166 (91.7)	166 (83.0)
Discontinuations	19 (9.5)	15 (8.3)	34 (17.0)
Reasons for discontinuation			
Adverse events	6 (3.0)	5 (2.8)	11 (5.5)
Consent withdrawal	9 (4.5)	10 (5.5)	19 (9.5)
Physician's decision	4 (2.0)	0 (0.0)	4 (2.0)

Note

Total numbers of patient evaluated are 200 during 4‐wk switching period, 181 during 4‐wk post‐switch period, and 200 during 8‐wk assessment period.

For 82 patients who had used aripiprazole as the primary antipsychotic, the discontinuation rate at week 8 was 4.9%. In addition, 1.2% of these patients discontinued due to adverse events. In contrast, the discontinuation rate at week 8 was 25.4% for 118 patients who used non‐aripiprazole as primary antipsychotic. Of these patients, 8.5% discontinued treatment due to adverse events. Overall, discontinuation rates in patients who had used non‐aripiprazole as primary antipsychotic were generally as high as 20 to 30%, regardless of the primary antipsychotics (Table 3).

**TABLE 3:** Discontinuations by primary antipsychotic (during 8 wk)

Primary antipsychotic [mean total daily CP‐equivalent dose^b^, mg/d]	N	Discontinuations n (%)	Reasons for discontinuation n (%)		
			Adverse events	Consent withdrawal	Physician's decision
Aripiprazole [433.4]	82	4 (4.9)	1 (1.2)	3 (3.7)	0 (0.0)
Non‐aripiprazole	118	30 (25.4)	10 (8.5)	16 (13.6)	4 (3.4)
Olanzapine [597.3]	36	10 (27.8)	5	3	2
Risperidone [508.9]	33	8 (24.2)	2	6	0
Paliperidone [757.5]	16	4 (25.0)	1	2	1
Typical antipsychotics^a^	14	3 (21.4)	1	2	0
Blonanserin [268.8]	8	2 (25.0)	1	1	0
Quetiapine [578.3]	8	2 (25.0)	0	2	0
Perospirone [150.0]	3	1 (33.3)	0	0	1
Total	200	34(17.0)	11(5.5)	19(9.5)	4(2.0)

Abbreviations: CP, chlorpromazine; N, number of patients evaluated in each primary antipsychotic.

^a^ Haloperidol (5 patients, 267.7 mg/d), sulpiride (3 patients, 125.0 mg/d), bromperidol (2 patients, 225.0 mg/d), levomepromazine (2 patients, 87.5 mg/d), mosapramine (1 patient, 727.0 mg/d), and periciazine (1 patient, 270.0 mg/d).

^b^ Total dose of prior antipsychotics, including the primary drug, administered 30 d before informed consent.

When patients who had used aripiprazole as primary antipsychotic and those using non‐aripiprazole were compared by univariate logistic regression with discontinuation rate over the 8‐week assessment period as the response variable, odds ratio of 6.648 (*P* = .0006) was confirmed. The odds ratio of discontinuation rate by mean total daily CP‐equivalent dose of previous antipsychotics was 1.000 (*P* = .3970; Table 4).

**TABLE 4:** Univariate Logistic Regression for discontinuation rate during 8 wk (4‐wk switching Period + 4‐wk post‐switch period)

Factors	Odds ratio	95% confidence interval	*P* value^a^
Age (y)	0.991	0.966‐1.016	.4656
Baseline PANSS total score (in 1 point)	1.002	0.985‐1.018	.8558
Number of previous antipsychotics	1.013	0.566‐1.813	.9664
Total daily CP‐equivalent dose of previous antipsychotics (mg/d)	1.000	0.999‐1.001	.3970
Primary antipsychotic: aripiprazole or non‐aripiprazole	6.648	2.242‐19.710	.0006
Primary antipsychotic: olanzapine or non‐olanzapine	0.446	0.191‐1.041	.0619
Primary antipsychotic: risperidone or non‐risperidone	0.576	0.234‐1.416	.2296
Primary antipsychotic: paliperidone or non‐ paliperidone	0.584	0.177‐1.935	.3792

Abbreviations: CP, chlorpromazine; PANSS, Positive and Negative Syndrome Scale.

^a^ Wald test.

### 3.5 Adverse events

Adverse events reported with a ≥ 5% incidence during the 8‐week assessment period (4‐week switching phase + 4‐week post‐switch phase) were nasopharyngitis (13.5%), schizophrenia (9.0%), insomnia (6.5%), headache (5.5%), and akathisia (5.5%). Serious adverse events occurred in nine patients (4.5%; Table 5).

**TABLE 5:** Adverse events

	4‐wk switching period n (%)	4‐wk post‐switch period n (%)	8‐wk assessment period n (%)
Any adverse events	94 (47.0)	76 (42.0)	127 (63.5)
Serious adverse events	7 (3.5)	2 (1.1)	9 (4.5)
Adverse events reported with a ≥ 5% incidence			
Nasopharyngitis	19 (9.5)	11 (6.1)	27 (13.5)^a^
Schizophrenia	11 (5.5)	7 (3.9)	18 (9.0)
Insomnia	9 (4.5)	4 (2.2)	13 (6.5)
Akathisia	5 (2.5)	6 (3.3)	11 (5.5)
Headache	5 (2.5)	6 (3.3)	11 (5.5)

Note

Total numbers of patient evaluated are 200 during 4‐wk switching period, 181 during 4‐wk post‐switch period, and 200 during 8‐wk assessment period.

^a^ Three patients experienced adverse events during both the 4‐wk switching period and the 4‐wk post‐switch period.

Eleven patients discontinued treatment due to adverse events (MedDRA/J version 16.0 preferred terms): with schizophrenia in seven patients, akathisia in two patients, and thirst and oculogyric crisis, each in one patient (Table 6). Four of the 11 patients discontinued treatment due to serious adverse events (ie, schizophrenia in three patients and akathisia in one patient). Three of these patients received olanzapine, and one patient was administered haloperidol (Table 6). In the three patients previously treated with olanzapine, the onset of adverse events was day 28 (schizophrenia: stupor), day 29 (schizophrenia: soliloquy, hallucination auditory, delusional mood, feeling irritated, and confusion), and day 37 (akathisia: a Drug‐Induced Extrapyramidal Symptoms Scale [DIEPSS] akathisia score of 4, resulting in difficulty managing physical conditions), and in the patient previously treated with haloperidol was day 57 (schizophrenia: auditory hallucination, delusion, and silly smile). Therefore, the onset of serious adverse events in the four patients who discontinued due to a serious adverse event occurred around the end of the protocol‐specified antipsychotic switching (week 4) or 4 weeks after switching completion (week 8; Table 6).

**TABLE 6:** Eleven discontinuations due to adverse events

Primary antipsychotic (n)	Adverse event	Onset	Causality with the study drug	Severity	Serious (Yes/No)
Aripiprazole (82)	Oculogyric crisis	Day 3	Related	Mild	No
Olanzapine (36)	Thirst	Day 14	Related	Mild	No
	Schizophrenia	Day 29	Unrelated	Severe	Yes
	Schizophrenia	Day 28	Related	Severe	Yes
	Akathisia	Day 29	Related	Moderate	No
	Akathisia	Day 37	Related	Severe	Yes
Risperidone (33)	Schizophrenia	Day 15	Unrelated	Moderate	No
	Schizophrenia	Day 48	Related	Moderate	No
Paliperidone (16)	Schizophrenia	Day 45	Related	Mild	No
Blonanserin (8)	Schizophrenia	Day 12	Related	Mild	No
Haloperidol (5)	Schizophrenia	Day 57	Related	Severe	Yes

### 3.6 Psychiatric symptoms

The PANSS total score (mean ± SD) at the start of antipsychotic switching (baseline) was 69.8 ± 22.1 (N = 200), and mean change in PANSS total score from baseline to week 8 was −5.2 ± 10.9 (N = 168, observed cases). The change in PANSS total score from baseline to week 8 was compared between patients who had used aripiprazole as primary antipsychotic and those using another antipsychotic. In patients using aripiprazole, baseline PANSS total score was 64.6 ± 19.0 (N = 82) and the change from baseline to week 8 was −5.7 ± 10.0 (N = 78, observed case). In patients using non‐antipsychotic, baseline PANSS total score was 73.3 ± 23.5 (N = 118) and the change from baseline to week 8 was −4.7 ± 11.7 (N = 90, observed case). Therefore, there was no substantial change in PANSS total score from baseline to week 8 whether aripiprazole was used as the primary antipsychotic or not.

## 4 DISCUSSION

For patients who were switched to the antipsychotic brexpiprazole (a dopamine D_2_ receptor partial agonist), the discontinuation rate during the 8 weeks after treatment initiation was 4.9% for switches from a dopamine D_2_ receptor partial agonist (aripiprazole) and 25.4% for switches from dopamine D_2_ receptor antagonists (non‐aripiprazole). This result suggests that more clinical experience is needed to derive the best method for use when switching from dopamine D_2_ receptor antagonists to brexpiprazole.

Several studies have reported their results for the switch from dopamine D_2_ receptor antagonists to aripiprazole, a dopamine D_2_ receptor partial agonist like brexpiprazole. In the US‐BETA study conducted in the United States over an 8‐week assessment period, the rate of discontinuation for any cause was 35.0% and the rate of discontinuation due to adverse events was 17.1%. The adverse events with an occurrence of ≥ 10% were insomnia (24%), nausea (16%), headache (11%), and anxiety (10%).^10^ In the EU‐BETA study conducted in Europe over an 8‐week assessment period, the rate of discontinuation for any cause was 27.2% and the rate of discontinuation due to adverse events was 12.6%. Similarly, the adverse events with an occurrence of ≥ 10% incidence in the EU‐BETA study were insomnia (16.0%) and nausea (10.2%).^11^ Because of the differences in switching rules used in the present post hoc analysis (ie, for switching to brexpiprazole monotherapy), and the US‐BETA and EU‐BETA studies (ie, for switching to aripiprazole monotherapy), a limitation exists when comparing the results in the different studies. Nonetheless, we observed lower discontinuation rate and incidence of adverse events in the present study than those found in the above studies, suggesting that switching to brexpiprazole might be safer than switching to aripiprazole.

The discontinuation rate when switching to brexpiprazole may be further lowered when the following two points, (a) and (b), are considered.

### 4.1 (a) Taper period for the previous antipsychotic(s)

Brexpiprazole, a dopamine D_2_ receptor partial agonist, has a high affinity for the dopamine D_2_ receptor, serotonin 5‐HT_1A_ receptor, and serotonin 5‐HT_2A_ receptor, and low affinity for the muscarinic M_1_ receptor and histamine H_1_ receptor.^1^ When switching to brexpiprazole, the period required for tapering the previous antipsychotic(s), as well as the potential withdrawal phenomena that accompany the tapering or the discontinuation of the previous antipsychotic, should be scrutinized by the antipsychotic type or doses administered; this scrutiny should also be performed relative to the pharmacological properties of brexpiprazole. A panel of Italian experts in psychiatry suggests that switching to aripiprazole, which resembles brexpiprazole in pharmacology, should be performed by tapering olanzapine by 5 mg every 15 days or risperidone by 1 mg every 15 days, while scanning for the apparent withdrawal phenomena.^5^ These dose‐titration methods require at least one month to both taper and discontinue olanzapine or risperidone from the clinical doses usually administered. British experts have also proposed that the previous antipsychotic(s) should be carefully tapered over one to two months if there are plans to switch to aripiprazole monotherapy.^6^ Although there are no established guidelines for down‐titrating the previous antipsychotic(s) when switching to brexpiprazole, the former should be tapered over at least one month (longer than the 2‐week tapering period used in the present study), based on clinical experience and reports where there was a switch to aripiprazole, an antipsychotic with similar pharmacological properties to brexpiprazole.

### 4.2 (b) Period of concomitant treatment with previous antipsychotics and the effective dose (2 mg/d) of brexpiprazole

To avoid the deterioration of psychiatric symptoms during antipsychotic switching, it is recommended that plateau switch strategies be adopted to establish an effective blood concentration of the new antipsychotic while the previous antipsychotic is being tapered.^4^ Plateau switch strategies are especially adequate when there is a switch to aripiprazole which takes a relatively long period (ie, 14 days) before reaching steady state.^4^ Since brexpiprazole reaches steady state within 10 days after treatment initiation^12^, a plateau switch strategy where it is up‐titrated to the effective dose (2 mg/day) while the previous antipsychotic is being tapered may prevent the deterioration of psychiatric symptoms associated with antipsychotic switching. Plateau switch strategies, however, involve a cross‐titration period where there is an overlap between brexpiprazole and the previous antipsychotic. This exposes the patient to high doses of antipsychotics. Therefore, if plateau switch strategies are applied to the switching to brexpiprazole, the possible development of adverse events, such as sedation or extrapyramidal symptoms, should be carefully monitored.

In this analysis, four patients discontinued treatment due to serious adverse events which were severe, three of which were switched from olanzapine. This suggests the need for more careful switching procedures (eg, a slower down‐titration of previous antipsychotic(s)) than the schedule adopted in the present study for patients who were previously using olanzapine as their primary antipsychotic. If previous treatment contains antipsychotics with anticholinergic and antihistaminic effects, such as olanzapine, their receptor‐binding profiles should also be considered before a patient is switched to brexpiprazole.

Based on the present study, the dose of brexpiprazole could be increased from 1 mg/day up to 4 mg/day, if the patient achieved a CGI‐I score ranging from 3 to 7 and could tolerate the dose as decided by the investigator or sub‐investigator. In Japan, the currently approved brexpiprazole dose is 1 to 2 mg/day. Therefore, further evaluations are warranted to establish practical switching procedures that are consistent with this approved dose. As well, switching period of the study was 4 weeks. It is shorter than common switching strategy. In the further evaluation, switching period should be longer than this protocol. Regarding the observation period, the 4‐week duration of post‐switching phase is relatively short for the assessment of switching strategy. The results of discontinuation rate can reflect “initial” discontinuation, and longer duration is needed to assess outcome of switching to brexpiprazole particularly for those who received olanzapine before the switching to brexpiprazole.

## CONFLICT OF INTEREST

JI received honorarium from Otsuka Pharmaceutical Co., Ltd. for providing advices or lectures as a medical advisor for this long‐term treatment study with brexpiprazole in Japanese patients with schizophrenia. TU, SI, YK, and SM are employees of Otsuka Pharmaceutical Co., Ltd.

## AUTHOR CONTRIBUTIONS

JI, TU, YK, and SM contributed to the study design. YK contributed to the establishment of the analysis plan and the implementation of data analysis. JI, TU, and SI wrote a first draft of the manuscript. All authors contributed to the interpretation of data, critically revised the manuscript, and approved the final manuscript for submission.

## ETHICAL APPROVAL

Each study site initiated patient enrollment after the institutional review boards (IRBs) reviewed and approved the study.

## INFORMED CONSENT

All patients provided written informed consent before participation in the study. Written consent for minors/dependents was obtained from their legal representatives (parental authorities, spouses, guardians, or any person equivalent to them), but a personal signed informed consent was also provided by these patients.

## Data Availability

The data that support the findings of this study are proprietary in nature and are not publicly available. However, the data are available on request from the corresponding author upon reasonable request.
